# Inter-muscle differences in modulation of motor evoked potentials and posterior root-muscle reflexes evoked from lower-limb muscles during agonist and antagonist muscle contractions

**DOI:** 10.1007/s00221-020-05973-x

**Published:** 2020-11-22

**Authors:** Akira Saito, Kento Nakagawa, Yohei Masugi, Kimitaka Nakazawa

**Affiliations:** 1 Center for Health and Sports Science, Kyushu Sangyo University, Matsukadai, Higashi-ku, Fukuoka, Japan; 2 Graduate School of Arts and Sciences, The University of Tokyo, Komaba, Meguro-ku, Tokyo, Japan; 3 Japan Society for the Promotion of Science, Kojimachi, Chiyoda-ku, Tokyo, Japan; 4 Faculty of Sport Sciences, Waseda University, Mikajima, Tokorozawa, Saitama Japan; 5 Institute of Sports Medicine and Science, Tokyo International University, Matoba, Kawagoe, Saitama Japan

**Keywords:** Corticospinal excitability, Posterior root-muscle reflex, Electromyography, Quadriceps femoris, Spinal cord stimulation

## Abstract

Voluntary contraction facilitates corticospinal and spinal reflex circuit excitabilities of the contracted muscle and inhibits spinal reflex circuit excitability of the antagonist. It has been suggested that modulation of spinal reflex circuit excitability in agonist and antagonist muscles during voluntary contraction differs among lower-limb muscles. However, whether the effects of voluntary contraction on the excitabilities of corticospinal and spinal reflex circuits depend on the tested muscles remains unknown. The purpose of this study was to examine inter-muscle differences in modulation of the corticospinal and spinal reflex circuit excitabilities of multiple lower-limb muscles during voluntary contraction. Eleven young males performed isometric plantar-flexion, dorsi-flexion, knee extension, and flexion at low torque levels. Motor evoked potentials (MEPs) and posterior root-muscle reflexes from seven lower-leg and thigh muscles were evoked by transcranial magnetic stimulation and transcutaneous spinal cord stimulation, respectively, at rest and during weak voluntary contractions. MEP and posterior root-muscle reflex amplitudes of agonists were significantly increased as agonist torque level increased, except for the reflex of the tibialis anterior. MEP amplitudes of antagonists were significantly increased in relation to the agonist torque level, but those of the rectus femoris were slightly depressed during knee flexion. Regarding the posterior root-muscle reflex of the antagonists, the amplitudes of triceps surae and the hamstrings were significantly decreased, but those of the quadriceps femoris were significantly increased as the agonist torque level increased. These results demonstrate that modulation of corticospinal and spinal reflex circuit excitabilities during agonist and antagonist muscle contractions differed among lower-limb muscles.

**Electronic supplementary material:**

The online version of this article (10.1007/s00221-020-05973-x) contains supplementary material, which is available to authorized users.

## Introduction

Voluntary muscle contraction facilitates the excitability of corticospinal tract and spinal reflex circuits of the contracted muscle. Modulations of corticospinal tract and spinal reflex circuit excitabilities during voluntary contraction have been addressed using transcranial magnetic stimulation (TMS) and Hoffmann-reflex (H-reflex) techniques, respectively. Previous studies demonstrated that facilitation of corticospinal tract and spinal reflex circuit excitabilities during voluntary contraction differed between extensor and flexor muscles around the ankle joint (Nielsen et al. 1993; Morita et al. 2000; Geertsen et al. 2010). It has been suggested that corticospinal tract and spinal reflex circuit excitabilities are modulated differently in different muscles, which may be due to differences in physiological characteristics among the muscles, such as the density of muscle spindles within a muscle (Banks 2006) and the corticospinal projections onto the motoneurons of lower-limb muscles (Brouwer and Ashby 1992).

It is well known that the spinal reflex circuits of antagonist muscles are inhibited by activation of agonist muscles, which is commonly referred to as reciprocal inhibition (Nielsen 2016). More specifically, Ia afferents from agonist muscles inhibit the motoneurons of antagonist muscles through Ia inhibitory interneurons (Hultborn et al. 1971). Moreover, inhibitory interneurons are modulated during voluntary contraction, which would be caused by the descending drive and sensory inputs onto the interneurons (Shindo et al. 1984; Crone et al. 1987). For example, the H-reflex response of SOL was depressed during dorsi-flexion and the amount of H-reflex depression changed depending on TA muscle activity (Shindo et al. 1984; Nielsen and Kagamihara 1993; Nielsen et al. 1993). Although reciprocal inhibition has been studied for the lower-leg muscles, the modulation of spinal reflex circuits between knee extensors and flexors has received little attention despite the importance of their functional roles in physical activities. The findings of reciprocal inhibition of thigh muscles would be useful for understanding knee joint stability for reducing risks of knee joint injuries (Solomonow and Krogsgaard 2001). Previous studies suggested that reciprocal inhibition is weaker in vastus lateralis (VL) and vastus medialis than in long head of biceps femoris (BF) and semimembranosus muscles (Bayoumi and Ashby 1989; Hamm and Alexander 2010). Therefore, modulation of the spinal reflex circuit excitability of quadriceps femoris may be weaker than that of the hamstrings during their antagonist activation. However, whether the effects of voluntary contraction on the spinal reflex circuit excitabilities of antagonist muscles differ among the lower-limb muscles is unclear. To characterize inter-muscle differences in modulation of the monosynaptic spinal reflex circuits of the lower-limb, this study used transcutaneous spinal cord stimulation (tSCS) as a measure of the posterior root-muscle reflexes from multiple muscles in the lower-limb (Minassian et al. 2007). The tSCS mainly activates the posterior root in the spinal cord, and the evoked responses appear to have characteristics similar to the H-reflex (Courtine et al. 2007).

Regarding the corticospinal tract pathway, some previous studies showed the facilitation effects on the motor evoked potentials (MEPs) of antagonist muscles by TMS during voluntary contraction (Valls-Sole et al. 1994; Izumi et al. 2000; Geertsen et al. 2010). It is suggested that cortical facilitatory inputs spread between agonist and antagonist muscle pairs during voluntary contraction. Furthermore, the enhancement of MEP amplitude was different between tibialis anterior (TA) and soleus (SOL) muscles during their antagonist activation (Valls-Sole et al. 1994). These results suggest that facilitation of corticospinal tract excitability of the antagonists would differ among lower-leg muscles during voluntary contraction. However, the modulation of corticospinal tract excitability of quadriceps femoris and hamstrings during their antagonist activation has not been addressed. These modulations have been considered to result from specific modulation of spinal reflex circuit excitabilities of quadriceps femoris and hamstring muscles during antagonist activation. Therefore, this study assumed that MEP modulation differs between quadriceps femoris and hamstrings during their antagonist activation, and the modulation of antagonist muscles of the lower-limb during voluntary contraction may be clarified.

The purpose of this study was to examine inter-muscle differences in modulation of corticospinal tract and spinal reflex circuit excitabilities of multiple muscles in the lower-limb during isometric voluntary contractions. We hypothesized that modulation of both MEPs and posterior root-muscle reflexes would differ among lower-limb muscles during agonist and antagonist voluntary muscle contractions.

## Methods

### Subjects

This study included 11 male participants, whose physical characteristics were age 26.0 ± 2.8 years; height 174.9 ± 4.8 cm; and weight 67.3 ± 7.2 kg. The procedure, purpose, risks, and benefits associated with this study were explained to the subjects, and written informed consent was obtained from all of them. The ethics review committee on experimental research with human subjects of the Graduate School of Arts and Sciences at The University of Tokyo approved the experimental protocols, which were conducted in accordance with the guidelines of the Declaration of Helsinki.

### Isometric contraction tasks

Experiments were conducted while subjects were in the supine position on the bench of a dynamometer (CON-TREX, CMV AG, Dübendorf, Switzerland). This was because preferential recruitment of the sensory fibers was induced by tSCS in the supine position compared to the prone and standing positions (Danner et al. 2016). In the supine position, the hip joints were fully extended, and the right knee joint was flexed to 30°. The right ankle joint was plantar-flexed to 10° to relax the dorsi-flexor muscles and was fixed to the attachment of the dynamometer with non-elastic straps.

Following several warm-up trials, the subjects performed an isometric maximal voluntary contraction (MVC) for approximately 3 s. Isometric voluntary contraction types were plantar-flexion, dorsi-flexion, knee extension, and knee flexion. Two MVC trials were performed to obtain the maximal torque. The inter-trial interval was set to 1 min. Following a 2-min rest interval, weak voluntary contractions were performed at three torque levels (i.e., 5%, 10%, and 20% of MVC), and the order of these targets was randomized. The subjects were provided visual feedback of 10-Hz lowpass-filtered torque signals and the target torque level via a computer monitor using specific software (LabChart 7, ADInstruments, Melbourne, Australia).

### Surface EMG recording

Surface EMG signals were recorded from SOL, medial head of gastrocnemius (MG) and lateral head of gastrocnemius (LG), TA, rectus femoris (RF), VL, and BF in the right leg. Ag–AgCl electrodes (Vitrode F-150S, Nihon Kohden, Tokyo, Japan) with an inter-electrode distance of 20 mm were used for EMG acquisition from each muscle. The amplifier was set to a gain of 1000-fold with a bandpass filter between 5 Hz and 1 kHz (AB-611 J, Nihon Kohden). The EMG signals and torque signals were simultaneously sampled at 4 kHz using an AD converter (PowerLab, ADInstruments) and stored on a personal computer using software (LabChart 7, ADInstruments).

### TMS

A double-cone coil (outside diameter of 110 mm) was placed over the leg area of the left motor cortex to obtain MEPs from the right lower-leg and thigh muscles by TMS (Magstim 200 stimulator, Magstim, Dyfed, UK). At the beginning of the measurements, the optimal stimulating site (i.e., “hot spot”) providing the largest amplitude for the SOL evoked response was identified. The head of each subject was secured on a head rest, and TMS coil position was maintained throughout the experiment. Next, the resting motor threshold (RMT) was determined while subjects rested quietly. The RMT was defined as the lowest stimulation intensity for which peak-to-peak amplitudes of MEP were larger than 50 μV for least three of five stimuli. The TMS intensity was set to 130% of the RMT of SOL (Abdelmoula et al. 2016). The stimulation intensity in this study was 77.8 ± 14.7% of maximal stimulator output. MEPs were evoked during the resting and weak voluntary contraction conditions, and ten stimuli were delivered in each condition.

### tSCS

Posterior root-muscle reflexes in the lower-limb were evoked using a constant current electrical stimulator with a single rectangular pulse of 1-ms duration (DS7A, Digitimer, Hertfordshire, UK). An anode (100 × 75 mm) was placed over the midline of the abdomen between the xiphoid process of the sternum and the umbilicus, and a cathode (50 × 50 mm) was placed on the midline of the back between the spinous processes of the upper-lumbar vertebrae. Since the stimulation electrode position of tSCS at different spinal levels affects recruitment of the evoked responses (Danner et al. 2011; Roy et al. 2012), in this study, cathodes were positioned where larger responses were evoked from all recorded muscles at any stimulation intensity, based on visual determination of the response magnitude (Masugi et al. 2016; Nakagawa et al. 2018; Saito et al. 2019). The cathode was placed between L1 and L2 in all subjects. To confirm that the responses were initiated in the sensory fiber based on suppression of the second response owing to post-activation depression (Andrews et al. 2015), a double-pulse stimulation with a 50-ms inter-pulse interval and with various intensities was delivered while the subject was resting (Courtine et al. 2007; Minassian et al. 2007). To evoke posterior root-muscle reflexes from multiple muscles in the lower-limb simultaneously, the strongest stimulation intensity was chosen, which can produce a large first response without a second response for seven lower-limb muscles (Supplemental Figure S1). The mean stimulation intensity for tSCS was 61.2 ± 12.7 mA. Under the resting and weak voluntary contraction conditions, ten stimuli were delivered.

### Maximum M-wave (M_max_)

M-wave responses in the lower-leg and quadriceps femoris during the rest condition were evoked by transcutaneous electrical stimulation. A single rectangular pulse stimulation with 1-ms pulse duration was delivered to the peripheral nerves by a constant current stimulator (DS7A, Digitimer). The M-waves of the plantar-flexors (i.e., SOL, MG, and LG) and the dorsi-flexor (i.e., TA) were evoked by electrical stimulation applied to the posterior tibial nerve and the branch of the common peroneal nerve, respectively. The electrical stimulation applied to the femoral nerve to evoke M-wave of the knee extensors (i.e., RF and VL). The stimulation intensity was gradually increased until no further increase was observed in M-wave amplitude from the tested muscles using 5-mA increments (Folland et al. 2008). The maximum M-wave response (M_max_) was evoked by setting the supramaximal stimulus intensity to 5 mA above the maximum for the experimental measurements (Supplemental Figure S2). The mean stimulation intensity for the M-wave for the plantar-flexors (i.e., SOL, MG, and LG), TA, and knee extensors (i.e., RF and VL) was 37.5 ± 16.2 mA, 31.5 ± 12.0 mA, 38.0 ± 13.9 mA, respectively. Due to anatomical limitation, the M-wave response of BF could not be evoked by electrical stimulation to the peripheral nerve.

### Data analysis

Peak-to-peak amplitudes of the MEPs and posterior root-muscle reflexes were calculated for each muscle. The amplitudes of MEPs and posterior root-muscle reflexes across 10 trials were averaged for each condition (i.e., rest and four types of contraction at three torque levels). The MEP and posterior root-muscle reflex amplitudes of SOL, MG, LG, TA, RF, and VL were normalized by the peak-to-peak amplitude of each M_max_. Differences in peak-to-peak amplitudes of MEPs and posterior root-muscle reflexes between resting and weak voluntary contraction conditions are expressed as changes from resting.

As the background EMG, the root-mean-square (RMS) in a 500-ms window just before the stimulation by TMS and tSCS was calculated (Duclay et al. 2011). The background EMG activity of each muscle was normalized to the corresponding amplitude of M_max_.

Since the level of background EMG activity during voluntary contraction affects the magnitudes of MEPs and posterior root-muscle reflexes, modulation of MEPs and posterior root-muscle reflexes might be related to the background EMG activity, rather than neural modulation of corticospinal tract and posterior root-muscle reflex circuits. The relationship between background EMG activity and the evoked response size represents the gain of MEP (Izumi et al. 2001) and the spinal reflex (Capaday and Stein 1986). To confirm the relationship between background EMG activity and MEP or posterior root-muscle reflex amplitudes evoked from agonist and antagonist muscles, this study plotted the normalized peak-to-peak amplitudes on the *y*-axis against the background EMG activity on the *x*-axis. Each plot included the data for all conditions and subjects.

### Statistical analysis

Normality of the data distribution was investigated using the Kolmogorov–Smirnov test. This study used non-parametric statistical tests if the distribution of data was partly non-Gaussian. The amplitudes of the first and second responses induced by a double-pulse stimulation were compared using the Wilcoxon signed-rank test. To test the effect of contraction intensity, MEP and posterior root-muscle reflex amplitudes of the lower-limb muscles were analyzed by the Friedman test. To compare the effect of muscles within a muscle group, MEP and posterior root-muscle reflex amplitudes of triceps surae (i.e., SOL, MG, and LG) were analyzed by the Friedman test and these of quadriceps femoris (i.e., RF and VL) were analyzed by the Wilcoxon signed-rank test. When a significant effect was found, the Wilcoxon signed-rank test was performed, and the *p* values for multiple comparisons were adjusted using the Bonferroni correction. Furthermore, the Friedman test was used to compare the background EMG (% of M_max_) and the change from resting of the MEP and posterior root-muscle reflex amplitudes. When a significant effect of muscle was found, the Wilcoxon signed-rank test was performed. The relationship between background EMG (% of M_max_) and MEP or posterior root-muscle reflex amplitudes was analyzed using Pearson’s linear regression analysis. Data are expressed as means ± standard error (SE) in the figures and as means ± SD in the text and table.

## Results

### Double-pulse stimulation of tSCS

It was confirmed that the responses were initiated in the sensory fiber using double-pulse stimulation (Supplemental Figure S3). The amplitudes of the second responses were significantly smaller than of the first responses in all tested muscles (SOL: *p* = 0.003, MG: *p* = 0.003, LG: *p* = 0.003, TA: *p* = 0.003, RF: *p* = 0.008, VL: *p* = 0.003, BF: *p* = 0.003). The second response amplitudes relative to the first responses were, SOL: 5.0 ± 4.2%, MG: 5.7 ± 5.2%, LG: 5.2 ± 4.5%, TA: 9.8 ± 6.3%, RF: 51.2 ± 32.9%, VL: 31.7 ± 20.3%, and BF: 12.3 ± 10.8%.

### Background EMG activity

As shown in Table 1, the level of background EMG activity of the agonist muscles increased with increasing agonist torque levels. Significant effects of contraction intensity (*p* < 0.001) were observed in the level of background EMG activity of each agonist muscle. The background EMG activity of TA was significantly higher than that of SOL, MG, LG, RF, and VL muscles during their agonist contraction at each intensity (*p* < 0.05). As the antagonist muscles, significant effects of contraction intensity were observed in the background EMG activity of SOL, MG, LG, and TA during antagonist contractions (*p* < 0.05). The background EMG activity of TA was higher than that of SOL during their antagonist contraction at 5%, 10%, and 20%MVC levels (*p* < 0.05).

**Table 1:** Background EMG activity during weak voluntary contractions as agonist and antagonist

	Agonist			Antagonist		
Target torque	5%MVC	10%MVC	20%MVC	5%MVC	10%MVC	20%MVC
Mean RMS (% of M_max_)						
SOL	0.19 ± 0.09	0.26 ± 0.13	0.45 ± 0.21	0.05 ± 0.02	0.07 ± 0.04	0.08 ± 0.03
MG	0.17 ± 0.19	0.23 ± 0.24	0.41 ± 0.31	0.09 ± 0.06	0.12 ± 0.08	0.11 ± 0.07
LG	0.17 ± 0.15	0.24 ± 0.19	0.42 ± 0.30	0.10 ± 0.07	0.11 ± 0.09	0.12 ± 0.07
TA	0.72 ± 0.47*	1.00 ± 0.68*	1 .48 ± 0.91*	0.30 ± 0.42^†^	0.32 ± 0.36^†^	0.47 ± 0.51^†^
RF	0.16 ± 0.04	0.24 ± 0.05	0.52 ± 0.17	0.11 ± 0.07	0.13 ± 0.08	0.10 ± 0.04
VL	0.19 ± 0.11	0.26 ± 0.15	0.54 ± 0.32	0.11 ± 0.06	0.14 ± 0.07	0.13 ± 0.07

Data are mean ± SD. RMS each muscle was normalized by M_max_. Major agonist action of the muscles, plantar-flexion: SOL, MG, and LG; dorsi-flexion: TA; knee extension: RF and VL. Major antagonist action of the muscles, dorsi-flexion: SOL, MG, and LG; plantar-flexion: TA; knee flexion: RF and VL

*p < 0.05, vs. SOL, MG, LG, RF, and VL

^†^*p* < 0.05, vs. SOL

### MEP and posterior root-muscle reflex responses in agonist muscles

**Fig. 1:**
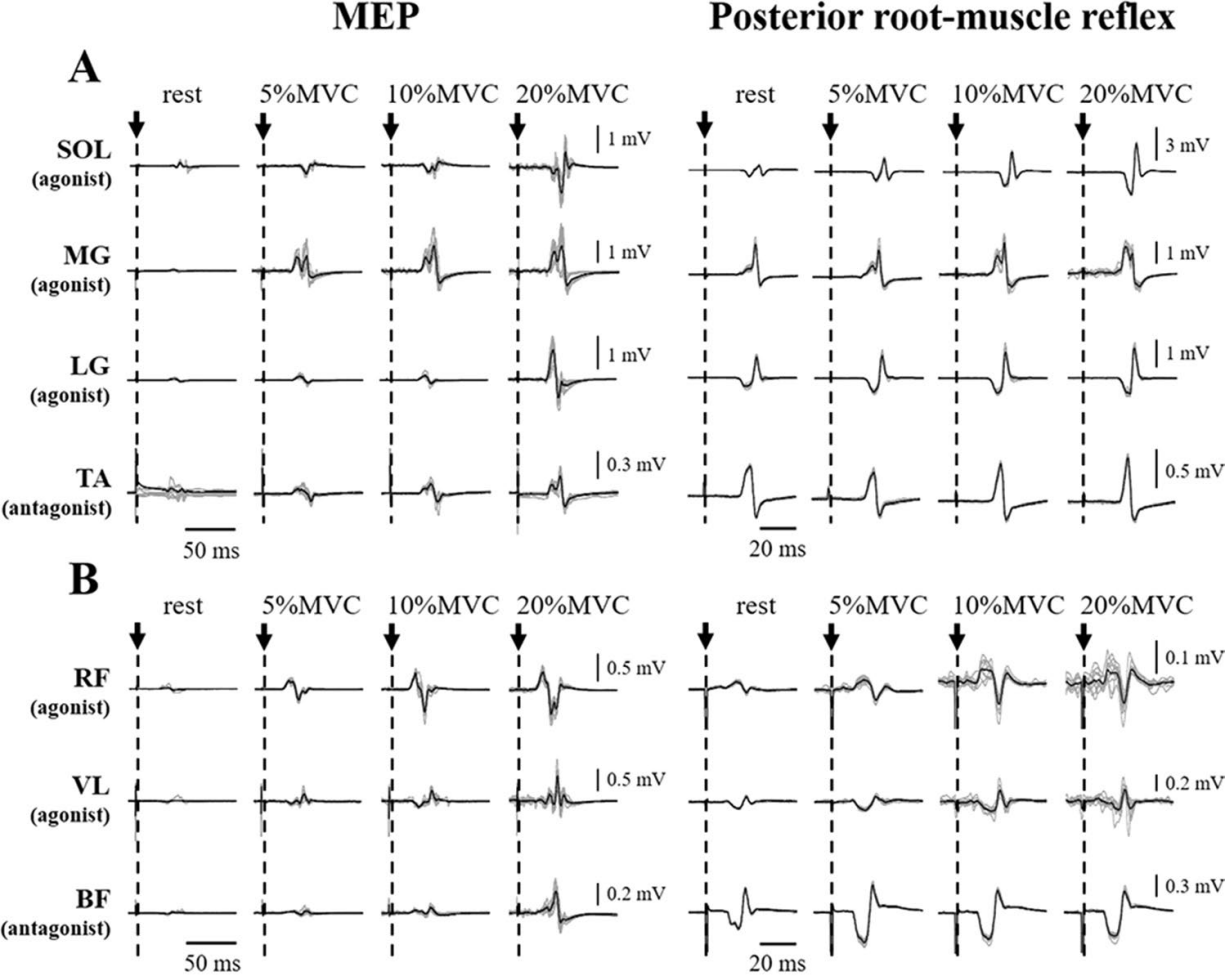
Typical example waveforms of MEPs and posterior root-muscle reflexes from a single subject. The MEPs and posterior root-muscle reflexes are induced during plantar-flexion (**a**) and knee extension (**b**). Four types of test conditions of the subject are the resting and weak contraction conditions at three different torque levels. Thin gray lines represent ten MEP and posterior root-muscle reflex waveforms overlaid at each trial, and the thick black line is the mean waveform of the MEP and posterior root-muscle reflex over ten trials. The filled black arrow and vertical dotted lines indicate the timing of the test stimulus by TMS and tSCS

**Fig. 2:**
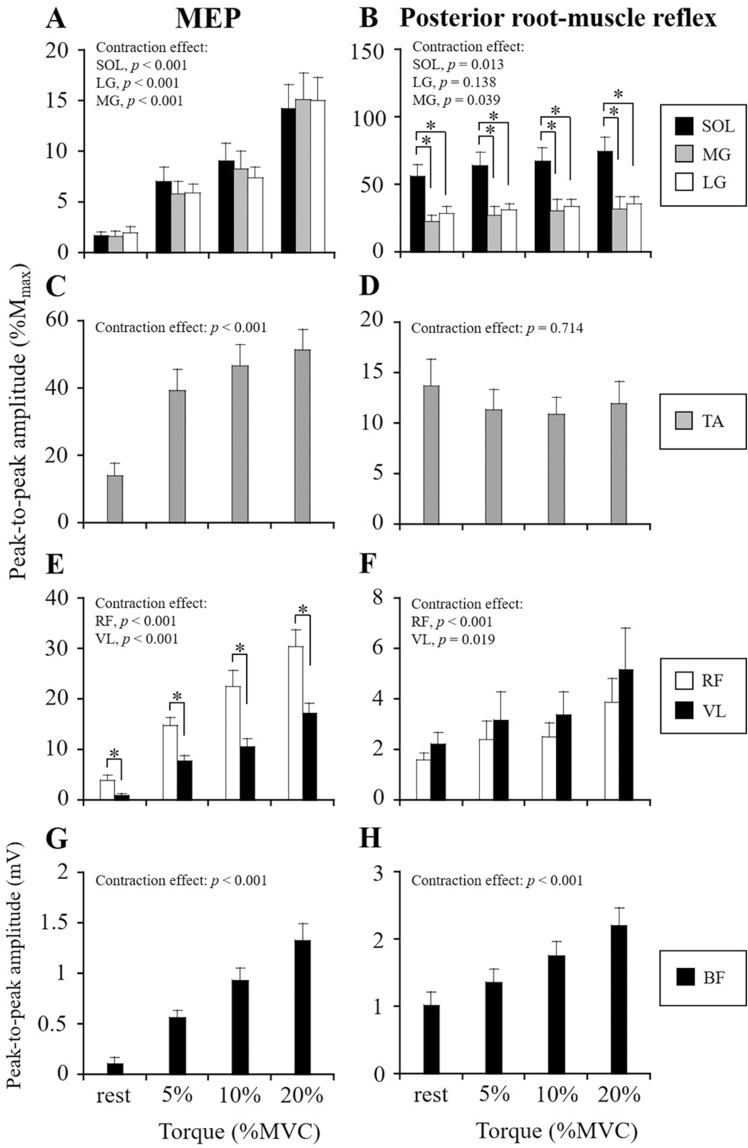
MEP (**a**, **c**, **e**, **g**) and posterior root-muscle reflex (**b**, **d**, **f**, **h**) amplitudes of agonist muscles during resting and weak voluntary contraction conditions. MEP and posterior root-muscle reflex amplitudes of triceps surae during plantar-flexion (**a**, **b**), TA during dorsi-flexion (**c**, **d**), quadriceps femoris during knee extension (**e**, **f**), and BF during knee flexion (**g**, **h**) are shown. Peak-to-peak amplitudes of MEPs and posterior root-muscle reflexes of each muscle are normalized by those of M_max_, except for BF. Values are expressed as means ± SE. *p < 0.05, significant difference between muscles

As shown in the typical waveforms of MEPs and posterior root-muscle reflexes (Fig. 1 and Supplemental Figure S4), voluntary contractions potentiated MEP and posterior root-muscle reflex amplitudes of agonist muscles. MEP amplitudes of each muscle were significantly increased with increasing agonist torque levels (*p* < 0.001, Fig. 2). The posterior root-muscle reflex amplitude of SOL (*p* = 0.013, Fig. 2b), LG (*p* = 0.039, Fig. 2b), RF (*p* < 0.001, Fig. 2f), VL (*p* = 0.019, Fig. 2f), and BF (*p* < 0.001, Fig. 2h) increased significantly with increasing agonist torque levels, whereas no facilitation of posterior root-muscle reflex was observed in MG (*p* = 0.138, Fig. 2b) and TA (*p* = 0.714, Fig. 2d). In the comparison between synergists within a muscle group, MEP amplitude was significantly higher for RF than for VL at each contraction intensity (*p* < 0.05, Fig. 2e). Posterior root-muscle reflex amplitude was significantly higher for SOL than for MG and LG at each contraction intensity (*p* < 0.05, Fig. 2b).

### MEP and posterior root-muscle reflex responses in antagonist muscles

**Fig. 3:**
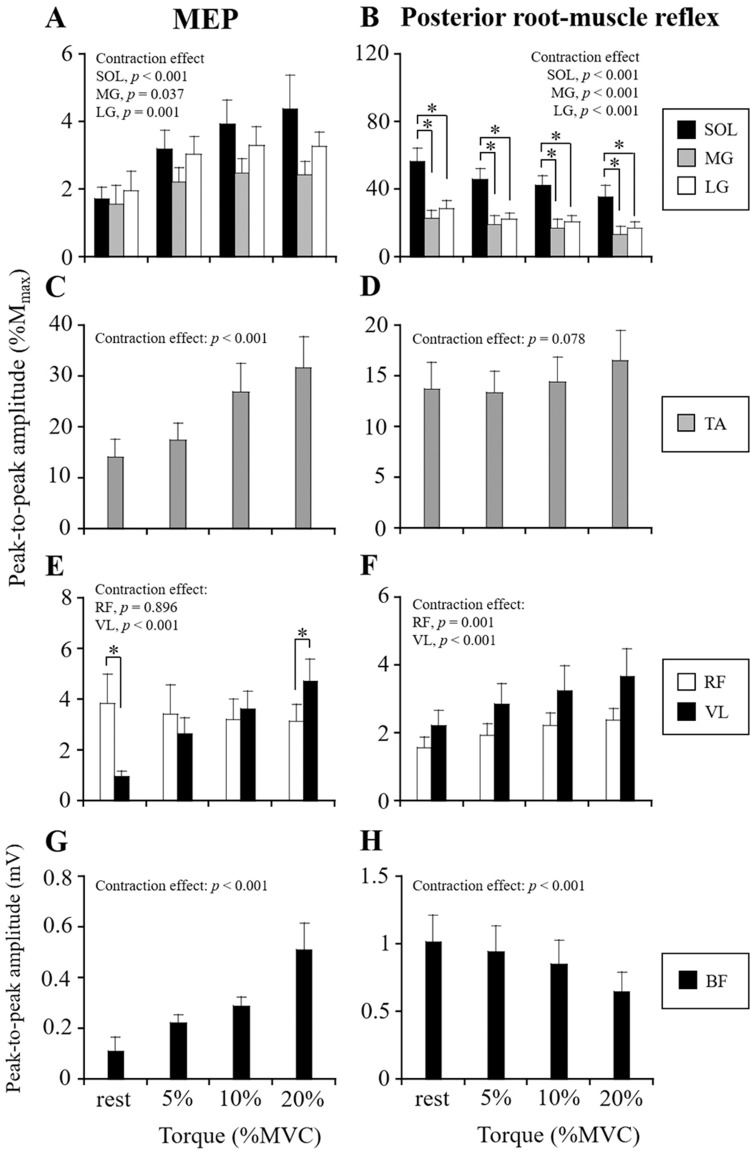
MEP (**a**, **c**, **e**, **g**) and posterior root-muscle reflex (**b**, **d**, **f**, **h**) amplitudes of antagonist muscles during resting and weak voluntary contraction. MEP and posterior root-muscle reflex amplitudes of triceps surae during dorsi-flexion (**a**, **b**), TA during plantar-flexion (**c**, **d**), quadriceps femoris during knee flexion (**e**, **f**), and BF during knee extension (**g**, **h**) are shown. Peak-to-peak amplitudes of MEPs and posterior root-muscle reflexes of each muscle are normalized by those of M_max_, except for BF. Values are expressed as means ± SE. *p < 0.05, significant difference between muscles

As shown in the typical waveforms of MEPs and posterior root-muscle reflexes (Fig. 1 and Supplemental Figure S4), voluntary contractions differently modulated MEP and posterior root-muscle reflex amplitudes of antagonist muscles. The MEP amplitudes of SOL (*p* < 0.001, Fig. 3a), MG (*p* = 0.037, Fig. 3a), LG (*p* = 0.001, Fig. 3a), TA (*p* < 0.001, Fig. 3c), VL (*p* < 0.001, Fig. 3e), and BF (*p* < 0.001, Fig. 3g) muscles increased significantly with increasing their agonist torque levels, but not the RF (*p* = 0.896, Fig. 3e). Posterior root-muscle reflex amplitudes of SOL (*p* < 0.001, Fig. 3b), MG (*p* < 0.001, Fig. 3b), LG (*p* < 0.001, Fig. 3b), and BF (*p* < 0.001, Fig. 3h) were significantly depressed as their agonist torque levels increased. Conversely, the posterior root-muscle reflex amplitudes of RF and VL were significantly increased by the knee flexion torque level (*p* < 0.001, Fig. 3f). No significant facilitation of the posterior root-muscle reflex of TA was observed during plantar-flexion (*p* = 0.078, Fig. 3d). In the comparison between synergists within a muscle group, the MEP amplitude of RF was significantly higher than that of VL at rest, but the amplitude of RF was significantly lower than that of VL at the 20%MVC level (*p* < 0.05, Fig. 3e). Posterior root-muscle reflex amplitude of SOL was significantly higher than that of MG and LG at each contraction intensity (*p* < 0.05, Fig. 3b).

### Changes of MEPs and posterior root-muscle reflexes among muscles

**Fig. 4:**
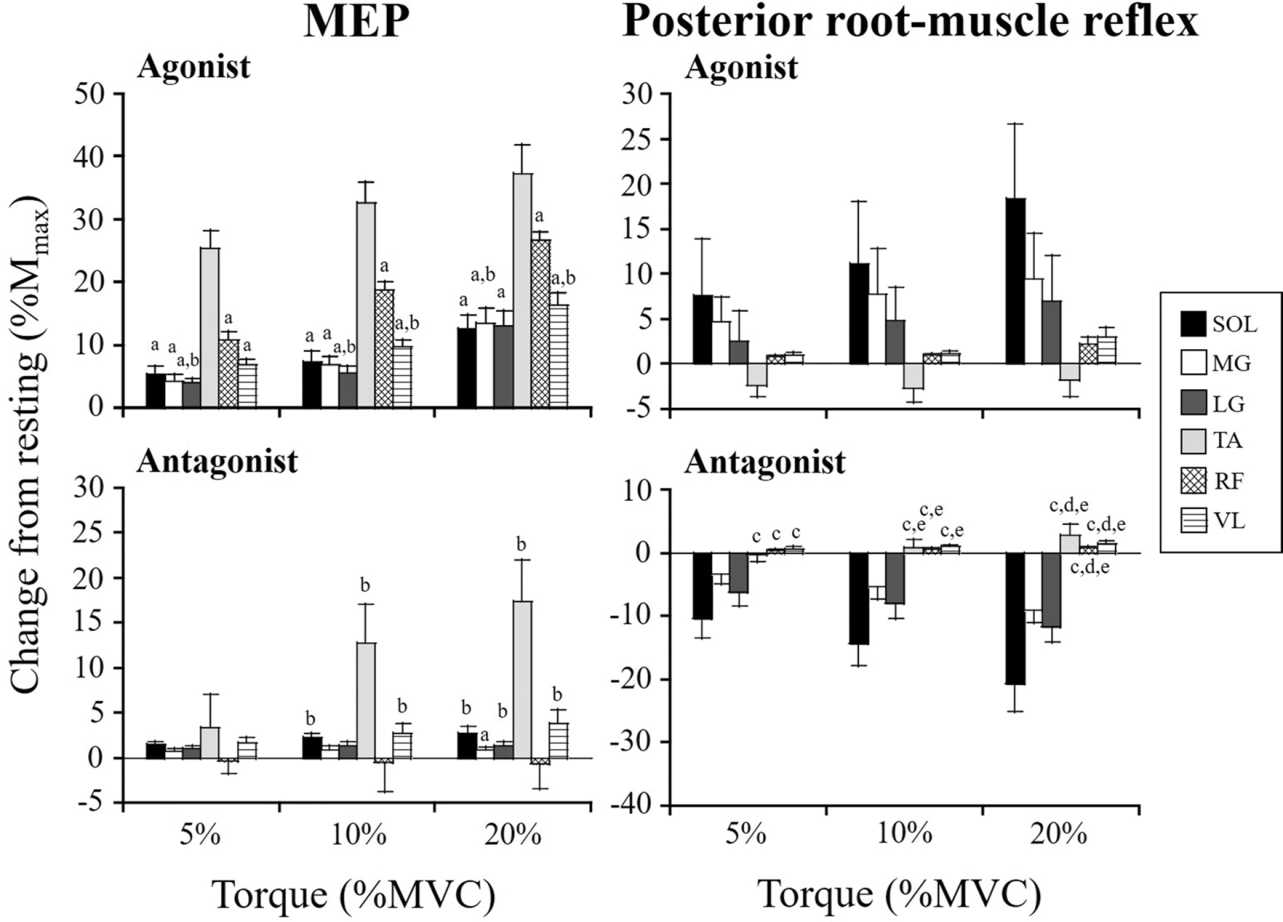
Changes from resting of MEP and posterior root-muscle reflex amplitudes of agonist and antagonist muscles. Values are expressed as means ± SE. **a** *p* < 0.05, vs. TA; **b** *p* < 0.05, vs. RF; **c** *p* < 0.05, vs. SOL; **d** *p* < 0.05, vs. MG; **e** *p* < 0.05, vs. LG

A significant effect of muscle was observed in MEPs of the change from resting of the agonist and antagonist muscles (*p* < 0.001, Fig. 4). Regarding the MEPs of agonist muscles, the change from resting was significantly higher for TA than for other muscles (*p* < 0.05), and that for RF was significantly higher than that for LG at 5% of MVC level, LG and VL at 10% of MVC level, and MG and VL at 20% of MVC level (*p* < 0.05). Regarding the MEPs of antagonist muscles, the change from resting was significantly higher for SOL, LG, TA, or VL than for RF at 10% and 20% MVC levels (*p* < 0.05). Regarding the posterior root-muscle reflexes of antagonist muscles, the changes from resting for SOL, MG, or LG were significantly lower than for TA, RF, and VL (*p* < 0.05).

### Relationship between background EMG and MEP or posterior root-muscle reflex

Regarding the MEPs of agonist muscles, the MEP amplitudes of SOL, MG, LG, TA, RF, and VL were significantly correlated to their background EMG activities (*r* = 0.57–0.73, *p* < 0.001) (Supplemental Figure S5). Regarding the MEPs of antagonist muscles, no significant correlation between MEP amplitude and background EMG activity was observed (*r* =  − 0.11 to 0.28, *p* > 0.05). Regarding the posterior root-muscle reflexes of agonist muscles, the posterior root-muscle reflex amplitudes of SOL, RF, and VL were significantly correlated with their background EMG activities (*r* = 0.31–0.40, *p* < 0.05), but not MG, LG, and TA (*r* = 0.14–0.25, *p* > 0.05). Regarding the posterior root-muscle reflexes of antagonist muscles, a significant correlation between the posterior root-muscle reflex amplitudes and background EMG activity was observed in SOL (*r* =  − 0.43, *p* = 0.005), but not MG, LG, TA, RF, and VL (*r* =  − 0.03 to 0.15, *p* > 0.05).

## Discussion

The purpose of this study was to examine inter-muscle differences in modulation of corticospinal tract and spinal reflex circuit excitabilities of multiple muscles in the lower-limb during isometric voluntary contractions. We hypothesized that modulation of both MEPs and posterior root-muscle reflexes would differ among lower-limb muscles during agonist and antagonist voluntary muscle contractions. The main findings of this study were that MEP modulation of TA was larger than that of triceps surae and quadriceps femoris during voluntary contraction, and the modulation of MEPs and posterior root-muscle reflexes of antagonist muscles differed among lower-limb muscles, which supported our hypothesis.

### Modulation of MEPs and posterior root-muscle reflexes of agonist muscles

The MEP and posterior root-muscle reflex amplitudes of agonist muscles were increased related to the torque level, except the posterior root-muscle reflex of TA (Fig. 2). In the ankle muscles, MEP and H-reflex amplitudes of SOL increased with increases in plantar-flexor torque, and the MEP amplitude of TA increases, but enhancement of the TA H-reflex was not obtained, during dorsi-flexion (Morita et al. 2000). In addition, MEP and H-reflex amplitudes of quadriceps femoris were larger with increasing isometric knee extension torque levels (Doguet and Jubeau 2014; Temesi et al. 2014). In particular, the change in MEP amplitude of RF was larger than of VL during knee extension (Temesi et al. 2014). Therefore, the results of this study support these previous studies.

This study demonstrated that the effects of voluntary contraction on MEP and posterior root-muscle reflex amplitudes differed among lower-limb muscles (Fig. 4). The spinal motoneurons mainly receives the excitatory inputs through both corticospinal and Ia afferent pathways. Hence, this study addressed the relationship between the background EMG activity level and MEP and posterior root-muscle reflex amplitudes of the tested muscles. Although there were differences to greater or lesser degrees, background EMG activity was positively correlated with the MEP amplitudes of each muscle and the posterior root-muscle reflex amplitudes of SOL, RF, and VL. In particular, the level of background EMG activity was strongly correlated with the MEP amplitude of triceps surae and quadriceps femoris (*r* > 0.67). Therefore, the level of background EMG activity is one of the factors inducing differences in MEP and posterior root-muscle reflex modulation between muscles.

Different extents of modulation of MEP and posterior root-muscle reflex amplitudes among lower-limb muscles during voluntary contraction may be related to the physiological characteristics of each muscle. More specifically, the relative contributions of corticospinal projections or Ia afferent inputs onto the spinal motoneurons may depend on the muscle. A previous study suggested that corticospinal projections onto the spinal motoneurons were stronger in TA than in SOL, MG, VM, and BF (Brouwer and Ashby 1992). In addition, the level of background EMG activity of TA was the highest among the lower-limb muscles (Table 1). Consequently, the largest MEP facilitation among lower-limb muscles during voluntary contraction occurred in TA (Fig. 4). Moreover, the density of muscle spindles is greater within SOL and TA than within any of quadriceps femoris and the hamstring muscles (Banks 2006). Since the relative density of muscle spindles differs among lower-limb muscles, the facilitation of the pathway of spinal motoneurons from Ia afferent inputs during voluntary contraction may be dependent on the muscles. Although there was no significant difference among lower-limb muscles, posterior root-muscle reflex facilitation was greater for SOL than for RF and VL (Fig. 4). As one of the possible factors inducing no facilitation of TA during dorsi-flexion, it is considered that the posterior root-muscle reflexes evoked by tSCS reflect a monosynaptic spinal reflex circuit (Courtine et al. 2007; Minassian et al. 2007). It has been suggested that the TA stretch reflex includes a larger long-latency component (i.e., transcortical pathway) than a short-latency component (i.e., spinal pathway) (Petersen et al. 1998). Assuming that the neural connection between Ia afferent and TA motoneuron pools is relatively weak, the facilitation of TA spinal motoneuronal excitability induced by a voluntary contraction might be relatively smaller compared to the facilitation of corticospinal tract excitability.

### Modulation of MEPs and posterior root-muscle reflexes of antagonist muscles

The previous studies showed that the MEPs of antagonist muscles around the ankle joint are enhanced during voluntary contraction (Nielsen et al. 1993; Valls-Sole et al. 1994; Izumi et al. 2000; Geertsen et al. 2010), whereas the H-reflexes of the antagonists are depressed (Shindo et al. 1984; Crone et al. 1987). In terms of the mechanisms involved in the observed distinct modulation between corticospinal tract and spinal reflex circuit excitabilities of the antagonist, several neurophysiological factors have been proposed (Nielsen 2016; Latash 2018). It has been suggested that facilitation of corticospinal tract excitability of the antagonist during voluntary contraction is caused by the activation of corticospinal cells projecting onto both agonist and antagonist motoneuron pools (Nielsen et al. 1993; Geertsen et al. 2010). Greater MEP facilitation of TA than of the other lower-limb muscles when the TA muscle was activated as agonist and antagonist was found (Fig. 4). Thus, similar neural mechanisms (e.g., corticospinal projections onto spinal motoneuron and background EMG activity) may be involved in the greater facilitation of corticospinal tract excitability of TA than of other lower-limb muscles during voluntary contraction. Moreover, the depression of spinal reflex circuit excitability of the antagonist during voluntary contraction is caused by Ia inhibitory interneurons (Hultborn et al. 1971). Although the background EMG activity also affects the modulation of the posterior root-muscle reflexes of antagonist muscles, no relationship between background EMG activity and MEP or posterior root-muscle reflex amplitudes of the antagonists was observed in most of the tested muscles in this study. Therefore, inhibition of spinal reflex circuit excitability of triceps surae and BF muscles would be induced via Ia inhibitory interneurons during dorsi-flexion and knee extension, respectively (Figs. 3 and 4).

The MEP amplitude of quadriceps femoris during knee flexion was differently modulated between the synergists; that is, the MEP amplitude of VL was increased, but the MEP amplitude of RF was slightly depressed with increasing knee flexion torque level (Fig. 3e). From the anatomical viewpoint, RF is the only bi-articular muscle in quadriceps femoris and shows specific neuromuscular activation profiles compared to the other mono-articular vasti muscles (Watanabe et al. 2012; Ema et al. 2016). Interestingly, the activation of RF was lower during multi-joint leg extension (including activation of the hip extensors) than during single-joint knee extension (Ema et al. 2016; Maeo et al. 2018). Although it is unclear which neurophysiological factor affects corticospinal tract excitability of RF during isometric knee flexion, we consider that the inhibitory neural inputs onto the corticospinal tract pathway and/or spinal motoneurons of RF were involved to some extent in this study.

In contrast to the results for triceps surae and BF, the posterior root-muscle reflex amplitudes of quadriceps femoris were facilitated during their antagonist activation (Figs. 3 and 4). A previous study showed that tendon reflexes evoked in RF and VL muscles were facilitated during isometric knee flexion (Watanabe 2018). In addition, Hamm and Alexander (2010) showed that depression of the H-reflex amplitude in the hamstrings by conditioning stimulation to the femoral nerve was greater than that in quadriceps femoris by sciatic nerve stimulation. They demonstrated that the reciprocal inhibitory effects were bi-directional between quadriceps femoris and hamstring muscles, but the reciprocal inhibitory connection is weaker in quadriceps femoris than in hamstring muscles (Hamm and Alexander 2010). In this study, the level of the antagonist EMG activity in quadriceps femoris muscles was increased during knee flexion (Table 1), and an increase in the MEP amplitude of the antagonist was observed in VL during knee flexion (Fig. 3). Therefore, during antagonist activation of quadriceps femoris, the reciprocal inhibition may be counteracted by facilitating the posterior root-muscle reflex excitability by voluntary contraction.

### Limitations

The sizes of H-reflex and MEP responses would be affected by the level of background EMG activity during voluntary contraction (Nielsen and Kagamihara 1993; Morita et al. 2000). Thus, the level of background EMG activity should be taken into consideration for inter-muscle differences of modulations of MEPs and posterior root-muscle reflexes among lower-limb muscles. This study showed that higher background EMG activity was observed in the TA muscle than in the other lower-limb muscles during their agonist and antagonist activation (Table 1). Furthermore, it should be noted that non-physiological factors affect action potentials of the EMG signals (Farina et al. 2002, 2004). The possibility that the evoked responses by tSCS involve cross-talk from the other muscles has been reported (Courtine et al. 2007). Thus, inter-muscle differences in modulation of MEPs and posterior root-muscle reflexes of the TA may be changed if the level of background EMG activities was matched among lower-limb muscles during voluntary contraction.

Differences in the magnitudes of MEPs and posterior root-muscle reflexes between lower-limb muscles may affect the modulation of agonist and antagonist muscles during voluntary contraction. It should be considered that sensitivity was different among the lower-limb muscles to modulate MEPs and posterior root-muscle reflexes. This was because this study evoked MEPs and posterior root-muscle reflexes from multiple muscles in the lower-limb simultaneously by TMS and tSCS with one stimulating site, respectively. For example, some previous studies evoked MEPs from individual tested muscles by TMS of the motor cortex where the optimal stimulation site (i.e., hot spot) was determined for each muscle separately (Geertsen et al. 2010; Izumi et al. 2000). However, it was difficult to determinate individual hot spots of the lower-limb area of the motor cortex by TMS because of the narrow and limited area and the location deeper from the scalp. Furthermore, the sensitivity of the H-reflex to facilitation and inhibition is related to the size of the test H-reflex (Crone et al. 1987). For example, the posterior root-muscle reflex of SOL was larger but the reflexes of RF and VL were relatively smaller (Figs. 2, 3). Although the posterior root-muscle reflex of SOL was potentiated as well as the MG and LG during plantar-flexion (Fig. 2b), the amplitude of SOL in some subjects may reach to the plateau of the recruitment curve by test stimulation intensity of tSCS. This is a potential factor inducing insensitivity to increase of posterior root-muscle reflex. Regarding the posterior root-muscle reflexes of RF and VL, this study confirmed that the amplitudes of second responses were depressed than the first responses by a paired-pulse stimulation of tSCS. Though, the second response amplitudes relative to the first responses of RF and VL were higher than the other tested muscles (Supplemental Figure S3). Thus, the posterior root-muscle reflexes of RF and VL might be insensitive to modulate because these responses by tSCS would partly include the activation of the motor fibers. Therefore, the size of the MEP and posterior root-muscle reflex was considered one of the limitations in the comparison of modulation of MEP and posterior root-muscle reflex amplitudes among lower-limb muscles.

### Conclusion

This study concludes that modulation of MEPs and posterior root-muscle reflexes of both agonist and antagonist muscles during voluntary contraction differed among lower-limb muscles. The evidence for the specific modulation of corticospinal tract and spinal reflex circuit excitabilities of agonist and antagonist muscles was extended by combining TMS and tSCS techniques. In particular, the corticospinal tract and spinal reflex circuit excitabilities of quadriceps femoris muscles were modulated differently with triceps surae and hamstring muscles during their antagonist activation. Modulation of corticospinal tract and spinal reflex circuit excitabilities in agonist and antagonist muscles during voluntary contraction appears to depend on the physiological characteristics of the muscle.

## Electronic supplementary material

Below is the link to the electronic supplementary material.

Supplemental data: https://doi.org/10.6084/m9.figshare.12387380.v6 (PDF 233 KB)
