# Supplementary material for: Inter-muscle differences in modulation of motor evoked potentials and posterior root-muscle reflexes evoked from lower-limb muscles during agonist and antagonist muscle contractions
Source: Exp Brain Res. 2020 Nov 22;239(2):463–74. doi: 10.1007/s00221-020-05973-x (PMC7936942; doi:10.1007/s00221-020-05973-x)
Supplement: Supplementary file 1 — Supplemental data: https://doi.org/10.6084/m9.figshare.12387380.v6 (PDF 233 KB) [file 221_2020_5973_MOESM1_ESM.pdf]

Supplemental Information

Title: Inter-muscle differences in modulation of motor evoked potentials and posterior root-muscle reflexes evoked from lower-limb during agonist and antagonist muscle contractions

Akira Saito[1,2,3], Kento Nakagawa[4], Yohei Masugi[2,5], Kimitaka Nakazawa[2]

1. Center for Health and Sports Science, Kyushu Sangyo University, Matsukadai, Higashi-ku, Fukuoka, Japan
2. Graduate School of Arts and Sciences, The University of Tokyo, Komaba, Meguro-ku, Tokyo, Japan
3. Japan Society for the Promotion of Science, Kojimachi, Chiyoda-ku, Tokyo, Japan
4. Faculty of Sport Sciences, Waseda University, Mikajima, Tokorozawa, Saitama, Japan
5. Institute of Sports Medicine and Science, Tokyo International University, Matoba, Kawagoe, Saitama, Japan

Corresponding Author: Akira Saito, Ph.D.

Supplemental Figure S1

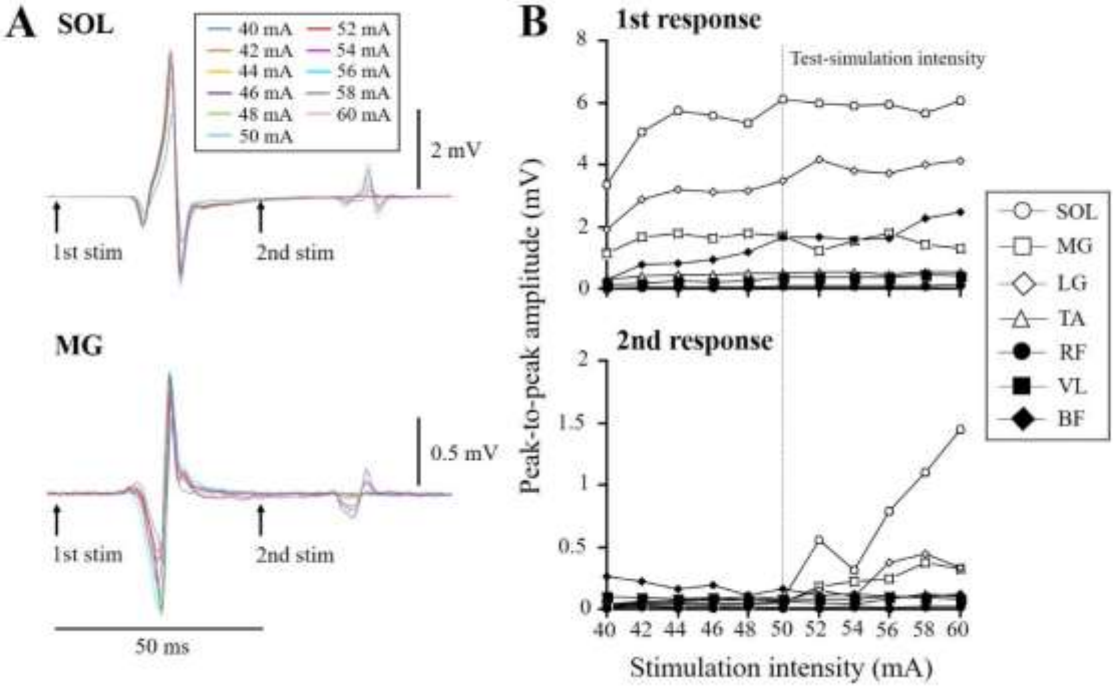

A typical example of stimulation intensity determination by tSCS with a double-pulse stimulation from a single subject. Posterior root-muscle reflex waveforms of SOL and MG when stimulation intensity ranges from 40 mA to 60 mA (A). Recruitment curves for the posterior root-muscle reflex amplitude of lower-limb muscles (B). Black dotted line indicates test stimulation intensity of tSCS. In this case, a stimulation intensity of 50 mA was chosen, which provides a large reflex on the first response, but the second response is as small as possible from all recorded muscles.

Supplemental Figure S2

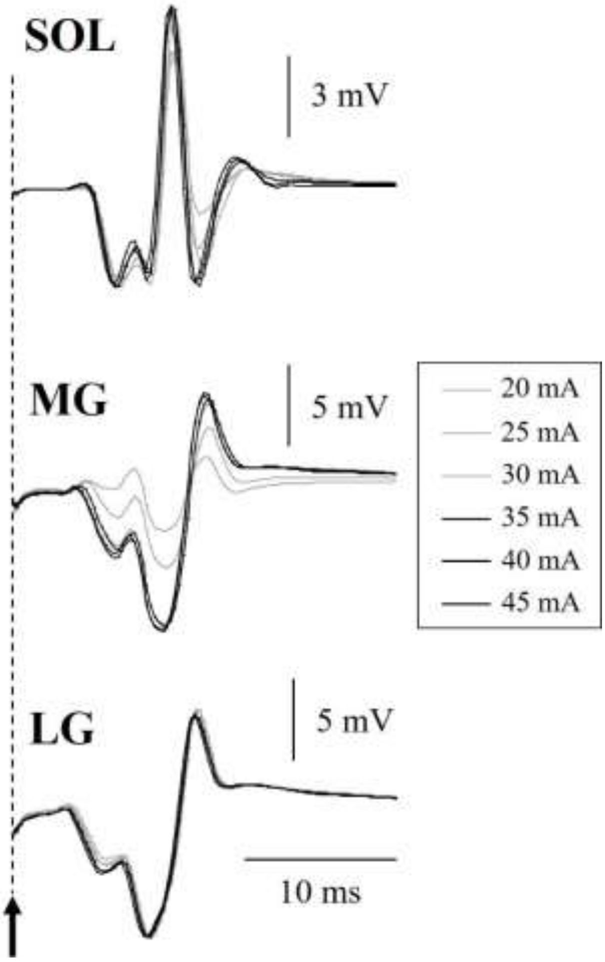

Typical example waveforms of the M-wave of SOL, MG, and LG muscles from a single subject. The stimulation intensity is gradually increased until no further increase is observed in M-wave amplitude from the tested muscles using 5-mA increments. The thick black lines represent the M-wave responses when electrical stimulation at supramaximal stimulus intensity is delivered. In this case, a stimulation intensity of 35 mA was chosen, which provides a large M-wave response and no further increase is observed in the tested muscles by the supramaximal stimulation. The filled black arrow and vertical dotted lines indicate the timing of the test stimulus by electrical stimulation to the posterior tibial nerve.

Supplemental Figure S3

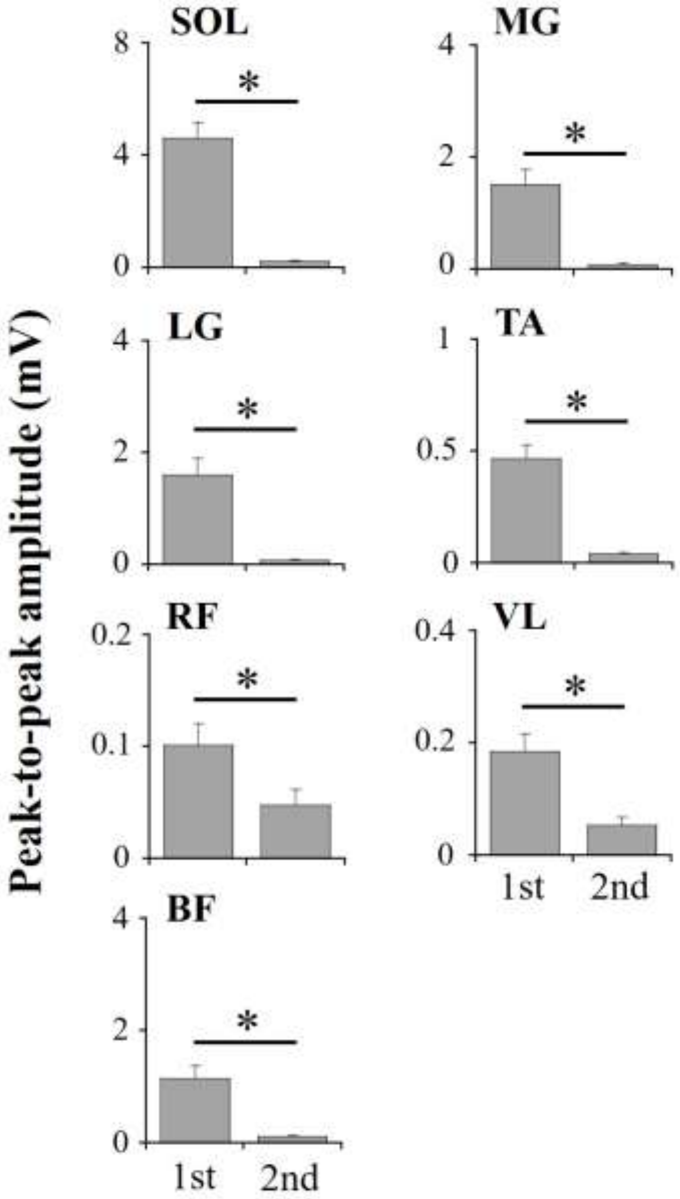

The amplitude of the responses of seven lower-limb muscles evoked by a double-pulse stimulation. Left and right bars represent first and second responses induced by tSCS for each muscle, respectively. Values are expressed as means ± SE. *: $p < 0.05$, significant difference between first and second responses

Supplemental Figure S4

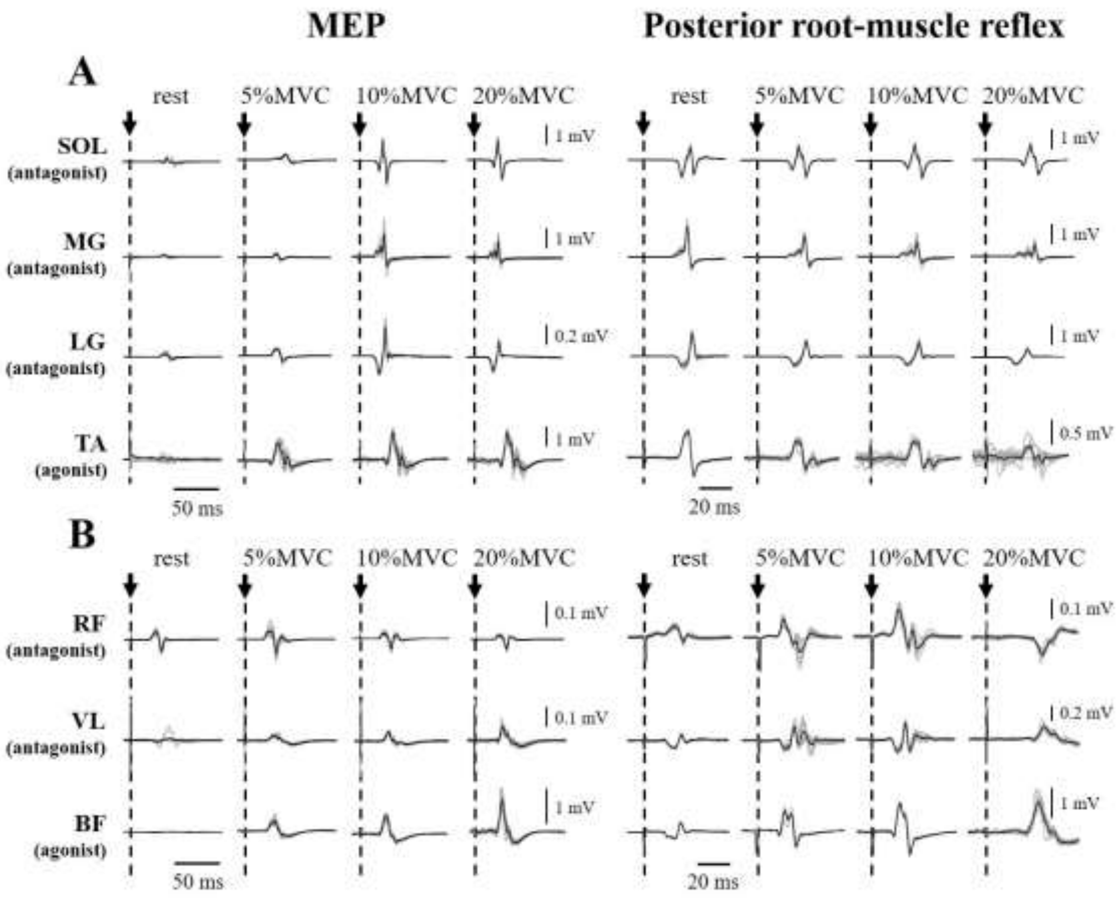

Typical example waveforms of MEPs and posterior root-muscle reflexes from a single subject. The MEP and posterior root-muscle reflex responses are induced during dorsi-flexion (A) and knee flexion (B). The 4 types of test conditions of the subject are the resting and weak contraction conditions at three different torque levels. The thin grey lines represent ten MEP and posterior root-muscle reflex waveforms overlaid at each trial, and the thick black line is the mean waveform of the MEP and posterior root-muscle reflex over 10 trials. The filled black arrow and vertical dotted lines indicate the timing of the test stimulus by TMS and tSCS.

Supplemental Figure S5

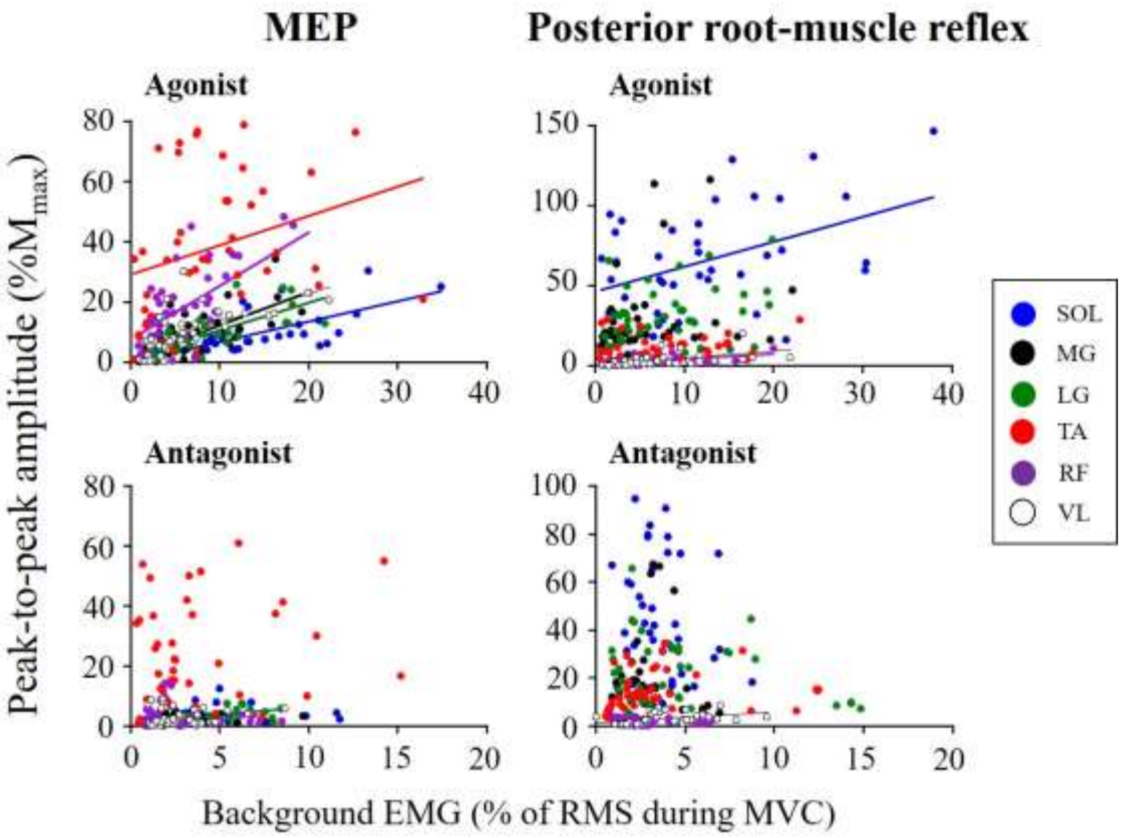

Relationships between background EMG and MEP or posterior root-muscle reflex amplitudes of agonist and antagonist muscles. Major agonist action of the muscles, plantar-flexion: SOL, MG, and LG; dorsi-flexion: TA; knee extension: RF and VL. Major antagonist action of the muscles, dorsi-flexion: SOL, MG, and LG; plantar-flexion: TA; knee flexion: RF and VL. The MEP and posterior root-muscle reflex amplitudes for at all conditions and subjects are plotted against the background EMG activity of each muscle. Each point represents the data for all subjects. Linear regression shows a significant linear relationship between background EMG activity and MEP or posterior root-muscle reflex amplitude.
